# The association between the planetary health diet index (PHDI) and muscular dystrophies: A mediating role of phenotypic age

**DOI:** 10.1097/MD.0000000000048612

**Published:** 2026-05-08

**Authors:** Xiaomei Lin, Shaoqun Huang, Hongyang Gong

**Affiliations:** a Department of Orthopedics, Fuzhou Hospital of Traditional Chinese Medicine Affiliated to Fujian University of Traditional Chinese Medicine, Fuzhou City, Fujian Province, China; b Department of Oncology Surgery, Fuzhou Hospital of Traditional Chinese Medicine Affiliated to Fujian University of Traditional Chinese Medicine, Fuzhou City, Fujian Province, China; c Department of Physiology, College of Medicine, Chosun University, Gwangju, Republic of Korea.

**Keywords:** mediation analysis, muscular dystrophies, NHANES, phenotypic age, planetary health diet index

## Abstract

To address the challenges posed by global aging and changing dietary habits, understanding the potential impact of healthy dietary patterns on diseases such as muscular dystrophies (MDs) and aging is crucial. The planetary health diet index (PHDI) is a dietary scoring system designed to balance human health and environmental sustainability by promoting the consumption of plant-based foods while reducing the intake of red meat, sugar, and highly processed foods. Additionally, phenotypic age is a composite measure based on multiple clinical biomarkers used to assess an individual’s biological age. This study examines the relationship between PHDI and MDs and evaluates whether phenotypic age moderates this association. This study conducted a cross-sectional analysis of 2951 participants from the 2011 to 2018 National Health and Nutrition Examination Survey database. Subgroup analysis, restricted cubic splines, and multivariable logistic regression were employed to investigate the association between PHDI and the prevalence of MDs. Additionally, mediation analysis was performed to explore potential associations between phenotypic age, PHDI, and MDs. A total of 2951 participants were included in this study, of whom 224 had MDs. After adjusting for all variables using multivariable logistic regression, each 10-point increase in PHDI was associated with a 15% reduction in MDs prevalence (odds ratio [OR] = 0.85, 95% confidence interval [CI]: 0.73–0.99), while each one-unit increase in phenotypic age was associated with a 7% increase in MDs prevalence (OR = 1.07, 95% CI: 1.01–1.14). Additionally, phenotypic age decreased as PHDI increased (β = ‐0.31, 95% CI: −0.53 to −0.10). Mediation analysis indicated that phenotypic age statistically accounted for a proportion of the observed association between PHDI and MDs in this cross-sectional population. Adherence to the planetary health diet guidelines may reduce the prevalence of MDs and slow biological aging. Furthermore, phenotypic age may partially account for the observed cross-sectional association between dietary quality and MDs, highlighting a potential link between diet, biological aging, and muscle health.

## 1. Introduction

Muscular dystrophies (MDs) are a group of genetic disorders characterized by progressive muscle weakness and degeneration, affecting millions worldwide.^[1,2]^ According to systematic reviews and meta-analyses, the overall prevalence of MDs is approximately 16.14 cases per 100,000 individuals (95% confidence interval [CI]: 11.21–23.23), with significant variation across geographic regions and subtypes.^[3]^ These disorders place a substantial burden on patients, families, and healthcare systems, often leading to decreased quality of life, increased disability, and premature death.^[4]^ In the United States, the treatment and care of MDs demand considerable economic investment, with annual costs for patients with Duchenne muscular dystrophy reaching $50,952. Additionally, these patients often require long-term medical care and adaptive housing modifications, further contributing to significant nonmedical costs.^[5]^ Given the progressive nature of MDs and the limited treatment options available, there is an urgent need to identify modifiable risk factors for prevention and develop targeted interventions that may potentially slow disease progression or improve patient outcomes.

Previous studies have explored the relationship between MDs and various dietary factors, emphasizing the potential role of nutrition in disease management and progression. For instance, research indicates that supplementing with protein and carbohydrates after endurance exercise can enhance muscle protein synthesis and reduce muscle protein breakdown in individuals with MDs, thereby improving muscle function and slowing muscle atrophy.^[6]^ Additionally, diets rich in antioxidants have been associated with reduced oxidative stress and improved muscle function in patients with Duchenne muscular dystrophy.^[7]^ However, the impact of overall dietary patterns on MD risk and progression remains unexplored. In this context, planetary health diet index (PHDI) has emerged as a promising tool for assessing dietary quality and its potential effects on health outcomes, including MDs. This index is based on the concept of the planetary health diet proposed by the EAT-Lancet Commission, which aims to promote human health and environmental sustainability.^[8]^ It encompasses various nutritional factors that may influence muscle health and disease progression, such as whole grains, fruits, vegetables, nuts, legumes, unsaturated fatty acids, and plant-based proteins. Investigating the relationship between PHDI and MDs could provide valuable insights into the role of integrated dietary patterns in the prevention and management of MDs, potentially offering new nutritional strategies for patients.

Biological aging is increasingly recognized as a key factor in the development and progression of various chronic diseases.^[9]^ Previous research has demonstrated an association between aging and an increased risk of low muscle mass, symptoms of which bear some resemblance to those of MDs.^[10]^ Moreover, dietary factors have been shown to influence the rate of biological aging, with healthier diets generally linked to a slower aging process.^[11]^ In this context, phenotypic age, a novel measure of biological age that integrates clinical and biochemical markers, offers a more accurate reflection of an individual’s aging rate and health status.^[12]^ Exploring the potential mediating role of phenotypic age in the relationship between PHDI and MDs may provide deeper insights into the mechanisms by which dietary patterns affect MD risk and progression. This approach could reveal new intervention pathways and assist in identifying individuals at high risk of developing or experiencing rapid progression of MDs. Utilizing data from the National Health and Nutrition Examination Survey (NHANES) from 2011 to 2018 to investigate these relationships offers several advantages, including a large, diverse, and nationally representative sample, standardized data collection protocols, and the availability of comprehensive dietary, clinical, and biochemical indicators.^[13]^ This robust dataset allows for a thorough examination of the complex interactions between diet, biological aging, and MDs, potentially providing valuable insights for clinical practice and public health interventions.

## 2. Methods

### 2.1. tudy participants

The NHANES, led by the Centers for Disease Control and Prevention, is a continuous cross-sectional survey designed to assess the health and nutrition status of the U.S. population. This survey employs a multistage, stratified, cluster sampling method to select representative samples nationwide, with data collection primarily consisting of interviews and physical examinations. The interview component covers demographic, socioeconomic, dietary, and health-related issues, while the physical examination includes physiological measurements and laboratory tests. During the survey, written informed consent is obtained from each participant to ensure voluntary participation and transparency. All data undergo standardized processing and quality control by the Centers for Disease Control and Prevention to ensure accuracy and consistency. The NHANES research project is reviewed and approved by the National Center for Health Statistics Research Ethics Review Board, ensuring the scientific integrity and ethical standards of the study.

This study is based on data from 4 NHANES cycles conducted between 2011 and 2018, employing stringent selection criteria to ensure sample representativeness and data integrity. Figure S1, Supplemental Digital Content illustrates the data selection process utilized in our research. Initially, 20,000 participants under the age of 20, as well as pregnant individuals, were excluded from the preliminary pool of 39,156 participants to ensure that the study focused on adults who were not pregnant, leaving a total of 22,370 individuals. Next, 4271 participants with incomplete PHDI data were excluded, resulting in 18,099 individuals. Subsequently, 14,272 participants with missing phenotypic age information were removed, reducing the sample size to 3827. Finally, 876 participants with incomplete muscular dystrophy data were excluded, yielding a final study sample of 2951 individuals. Through this series of rigorous selection steps, the high quality of the study sample and the reliability of the research findings were ensured.

### 2.2. MDs assessment

The existence of MD was the main finding of this investigation. For MD, NHANES does not offer a direct diagnostic variable. Rather, we used already recognized criteria in a recently published NHANES-based analysis (DOI: 10.3389/fneur.2025.1599600; DOI: 10.3389/fnut.2024.1465486). Appendicular lean mass (ALM) refers to the muscle weight of the limbs (arms and legs). It is typically measured using dual-energy X-ray absorptiometry, with data available from the NHANES examination dataset. The ALM/BMI ratio is widely regarded as an effective indicator for assessing muscle condition. Based on this metric, the diagnostic criteria for MDs are defined as follows^[1,14]^: an ALM/BMI ratio of <0.789 for males and <0.512 for females. This method not only considers muscle mass but also accounts for differences in body composition, thereby providing a more comprehensive and accurate assessment of muscle condition.

### 2.3. Definition of the PHDI

The PHDI is an emerging assessment tool developed by Cacau et al^[15]^ that aims to measure the alignment of individual diets with the health and sustainability guidelines established by the EAT-Lancet Commission. It emphasizes the crucial role of diet in supporting human health and environmental sustainability while encouraging increased consumption of plant-based foods and a reduction in the intake of animal-derived products. To evaluate the PHDI, we referenced several previously established calculation methods.^[16–18]^ PHDI comprises 14 components, categorized into 2 main domains: sufficiency and moderation, which assess recommended foods and those suggested for moderate consumption, respectively. *Sufficiency components*: this category includes foods such as seeds, legumes, whole grains, fruits, vegetables, nuts, unsaturated oils, and healthy foods. Each component is assigned a score ranging from 0 to 10, with higher scores indicating closer alignment with the recommended intake levels. *Moderation components*: this category emphasizes the reduction of foods that should be consumed in moderation. These include dairy products, poultry, eggs, fish, red meat, processed meats, saturated fats, trans fats, added sugars, and juices. Similar to the sufficiency components, these items are scored on a scale from 0 to 10, where higher scores indicate lower consumption of these foods. The total score for the PHDI ranges from 0 to 140, with higher scores indicating greater alignment with the EAT-Lancet dietary guidelines. The score for each component is calculated based on participants’ reported food intake and then compared to predetermined standards derived from the midpoint of the recommended intake ranges established by the EAT-Lancet Commission. The specific calculation method for the PHDI is detailed in Table S1, Supplemental Digital Content.

### 2.4. Definition of phenotypic age

According to previous studies,^[19]^ phenotypic age is a composite metric based on multiple biomarkers used to assess an individual’s biological age. The calculation process involves 9 clinical biomarkers, including white blood cell count, mean corpuscular volume, red blood cell distribution width, alkaline phosphatase, triglycerides, C-reactive protein (CRP), glucose, albumin, and creatinine, along with chronological age. After logarithmic transformation and standardization, these indicators are used to compute a “mortality risk score” via a Cox proportional hazards regression model. This score is then transformed onto an age scale to yield the phenotypic age.

The calculation formula is as follows:


$$\begin{array}{l} Phenotypic\;age\;=\;141.50\;+\;\frac{ln\left[-0.00553\times ln\left(1-xb\right)\right]}{0.09165} \end{array}$$

where: xb=−19.907 − 0.0336 × albumin + 0.0095 × creatinine + 0.0195 × glucose + 0.0954 × ln(CRP) − 0.0120 × lymphocyte percent + 0.0268 × mean cell volume + 0.3356 × red blood cell distribution width + 0.00188 × alkaline phosphatase + 0.0554 × white blood cell count + 0.0804 × chronological age.

### 2.5. Covariables

This study selected several covariates to comprehensively assess the potential impact of confounding factors. These covariates include age, sex, race, marital status, education level, family poverty income ratio (PIR), smoking, alcohol consumption, hypertension, diabetes, and self-reported hyperlipidemia. Detailed information about these covariates is provided in Table S2, Supplemental Digital Content.

### 2.6. Statistical analysis

To ensure that the data accurately represents the national population, all analyses employed sampling weights. The weight variable used in our study was the 2-day dietary sample weight (WTDR2D), and we calculated the new weight for the 2011 to 2018 period as 1/4 × WTDR2D.^[18]^ Continuous variables are presented as means ± standard deviations, while categorical variables are presented as frequencies (percentages). Weighted *t* tests were used for comparing continuous variables, and weighted Chi-square tests were applied for categorical variables. Weighted logistic regression was employed to explore the relationship between PHDI and MDs. Three logistic regression models were established: Model 1, which did not adjust for potential confounding factors; Model 2, which adjusted for covariates including age, sex, education level, marital status, PIR, and race; and Model 3, which further adjusted for smoking, alcohol consumption, hypertension, diabetes, and hyperlipidemia based on Model 2. Additionally, in Model 3, PHDI was treated as a continuous variable, and restricted cubic splines were utilized to illustrate the linear or nonlinear association between PHDI and MDs. Subsequently, stratified subgroup analyses were conducted based on Model 3 to explore potential variations in associations among subgroups.

Given the premise that “PHDI is statistically significantly associated with phenotypic age” and “phenotypic age is statistically significantly associated with muscular dystrophies (MDs),” mediation analysis was conducted to investigate whether the effect of PHDI on MDs is mediated by phenotypic age. The mediation effects were calculated using the “mediation” package in R software.^[20]^ Data processing was performed using R statistical software (version 4.3.1). A two-tailed *P*-value of <.05 was considered statistically significant.

## 3. Results

### 3.1. Baseline characteristics

This study analyzed 2951 samples, representing approximately 27.38 million individuals, and found a prevalence rate of 6% for MDs, which is closely associated with various demographic and socioeconomic factors. A higher prevalence of MDs was observed among males, married individuals, and the Mexican population. Furthermore, a higher education level and non-poverty status were associated with an increased risk of MDs. Notably, the MDs group exhibited a higher phenotypic age and a lower PHDI level compared to the non-MDs group. For more details, please refer to Table 1.

**Table 1:** Baseline characteristics of all participants were stratified by muscular dystrophies, weighted.

Characteristic	Overall, N = 27,386,966 (100%)	Nonmuscular dystrophies, N = 25,748,171 (94%)	Muscular dystrophies, N = 1,638,795 (6%)	*P* value
No. of participants in the sample	2951	2727	224	–
Age (%)				.215
20–30	10,728,238 (39%)	10,156,400 (39%)	571,838 (35%)	
31–40	9,142,314 (33%)	8,621,305 (33%)	521,009 (32%)	
>40	7,516,414 (27%)	6,970,467 (27%)	545,947 (33%)	
Gender (%)				.101
Male	13,483,999 (49%)	12,554,400 (49%)	929,598 (57%)	
Female	13,902,967 (51%)	13,193,771 (51%)	709,196 (43%)	
Race (%)				**<.001**
Other	5,525,976 (20%)	5054,289 (20%)	471,687 (29%)	
Non-Hispanic White	15,360,511 (56%)	14,802,889 (57%)	557,622 (34%)	
Non-Hispanic Black	3,029,667 (11%)	2,985,794 (12%)	43,872 (2.7%)	
Mexican American	3470,813 (13%)	2905,200 (11%)	565,614 (35%)	
Married/live with partner (%)				.217
No	10,898,982 (40%)	10,323,958 (40%)	575,024 (35%)	
Yes	16,487,984 (60%)	15,424,214 (60%)	1,063,770 (65%)	
Education level (%)				**<.001**
Below high school	2966,127 (11%)	2,544,638 (9.9%)	421,489 (26%)	
High school or above	24,420,839 (89%)	23,203,533 (90%)	1,217,306 (74%)	
PIR (%)				**.002**
Not poor	19,648,948 (77%)	18,704,615 (78%)	944,333 (65%)	
Poor	5889,902 (23%)	5373,044 (22%)	516,858 (35%)	
Smoking (%)				.935
Never	16,982,329 (62%)	15,955,399 (62%)	1,026,930 (63%)	
Former	5,018,357 (18%)	4,709,517 (18%)	308,840 (19%)	
Current	5,386,280 (20%)	5,083,256 (20%)	303,025 (18%)	
Drinking (%)				**.003**
Former	1,140,483 (4.4%)	1,048,146 (4.5%)	92,337 (6%)	
Heavy	7,231,997 (29%)	6,705,600 (28%)	526,397 (35%)	
Mild	8,606,498 (34%)	8,291,270 (35%)	315,228 (21%)	
Moderate	5811,183 (23%)	5,503,785 (23%)	307,398 (20%)	
Never	2,414,767 (9.6%)	2,144,153 (9.5%)	270,615 (18%)	
Hypertension (%)				**<.001**
No	22,451,567 (82%)	21,333,197 (83%)	1118,370 (68%)	
Yes	4,935,400 (18%)	4,414,975 (17%)	520,425 (32%)	
Diabetes (%)				**<.001**
No	25,862,113 (94%)	24,448,344 (95%)	1413,769 (86%)	
Yes	1,524,853 (6%)	1,299,828 (5%)	225,026 (14%)	
High cholesterol (%)				**.025**
No	22,825,898 (83%)	21,558,759 (84%)	1267,139 (77%)	
Yes	4,561,068 (17%)	4,189,412 (16%)	371,656 (23%)	
PHDI (mean [SD])	60.01 (15.47)	60.24 (15.49)	56.35 (14.78)	**.016**
PHDI (%)				.082
T1	9,131,412 (33%)	8,469,323 (33%)	662,088 (40%)	
T2	9,109,263 (34%)	8,526,121 (33%)	583,142 (36%)	
T3	9,146,291 (33%)	8,752,727 (34%)	393,564 (24%)	
Phenotypic age (mean [SD])	32.03 (9.78)	31.83 (9.74)	35.27 (9.86)	**<.001**
Phenotypic age (%)				**<.001**
T1	9,138,419 (34%)	8,813,254 (34%)	325,165 (20%)	
T2	9,131,639 (33%)	8,521,050 (33%)	610,589 (37%)	
T3	9,116,908 (33%)	8,413,867 (33%)	703,041 (43%)	

Mean (SD) for continuous variables: the *P* value was calculated by the weighted Students *T* test. Percentages (weighted N, %) for categorical variables: the *P* value was calculated by the weighted Chi-square test. Bold values indicate *P* < .05.

PHDI = planetary health diet index, PIR = ratio of family income to poverty, SD = standard deviations.

### 3.2. The association between PHDI and MDs

As shown in Table 2, we employed 3 different models to assess the relationship between PHDI and MDs. In Model 3, after adjusting for all variables, an increase of 10 points in PHDI was associated with a 15% reduction in the likelihood of developing MDs (odds ratio [OR]: 0.85, [95% CI: 0.73–0.99]). Additionally, in Model 3, the third quartile (T3) of PHDI was associated with a 46% reduction in the likelihood of developing MDs compared to the first quartile (T1) (OR: 0.54, [95% CI: 0.31–0.94]). The ORs consistently decreased from T1 to T3 with increasing PHDI, demonstrating a significant trend (*P* = .037). The results of Models 1 and 2 were similar.

**Table 2:** Association between PHDI, phenotypic age, and muscular dystrophies, NHANES 2011 to 2018.

Characteristics	Model 1 (OR [95% CI])	*P*-value	Model 2 (OR [95% CI])	*P*-value	Model 3 (OR [95% CI])	*P*-value
PHDI (muscular dystrophies)						
Continuous (per 10 scores)	0.85 (0.75–0.95)	.007	0.84 (0.73–0.96)	.016	0.85 (0.73–0.99)	.042
Tertile						
T1	1 (ref.)		1 (ref.)		1 (ref.)	
T2	0.87 (0.53–1.43)	.583	0.86 (0.51–1.47)	.567	0.86 (0.44–1.65)	.609
T3	0.58 (0.36–0.91)	.021	0.53 (0.32–0.87)	.015	0.54 (0.31–0.94)	.032
*P* for trend	.023		.016		.037	
Phenotypic age (muscular dystrophies)						
Continuous	1.04 (1.02–1.05)	<.001	1.08 (1.04–1.13)	.001	1.07 (1.01–1.14)	.022
Tertile						
T1	1 (ref.)		1 (ref.)		1 (ref.)	
T2	1.94 (1.22–3.09)	.007	3.11 (1.63–5.92)	.002	3.22 (1.56–6.65)	.005
T3	2.26 (1.50–3.42)	<.001	4.54 (1.86–11.12)	.002	3.62 (1.20–10.90)	.026
*P* for trend	<.001		.003		.024	

Model 1: no covariates were adjusted. Model 2: age, gender, education level, marital, PIR, and race were adjusted. Model 3: age, gender, education level, marital, PIR, race, smoking, drinking, hypertension, diabetes, and high cholesterol were adjusted.

CI = confidence interval, OR = odds ratio, PHDI = planetary health diet index, PIR = ratio of family income to poverty, NHANES = National Health and Nutrition Examination Survey.

**Figure 1.**
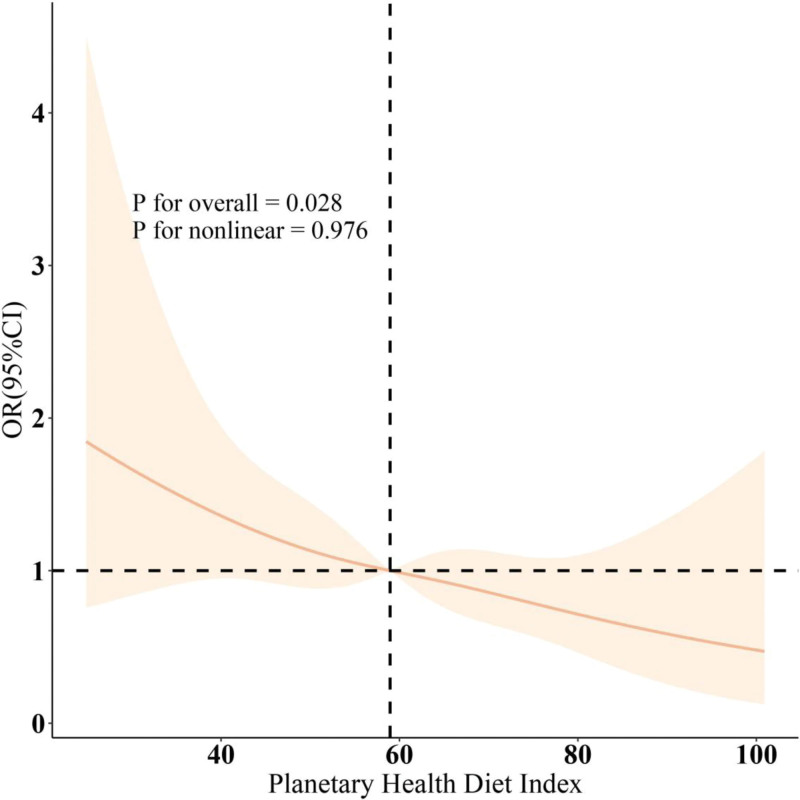
Dose–response relationships between PHDI and muscular dystrophies. OR (solid lines) and 95% confidence levels (shaded areas) were adjusted for age, gender, education level, marital, PIR, race, smoking, drinking, hypertension, diabetes, and high cholesterol. OR = odds ratio, PHDI = planetary health diet index, PIR = family poverty income ratio.

**Figure 2.**
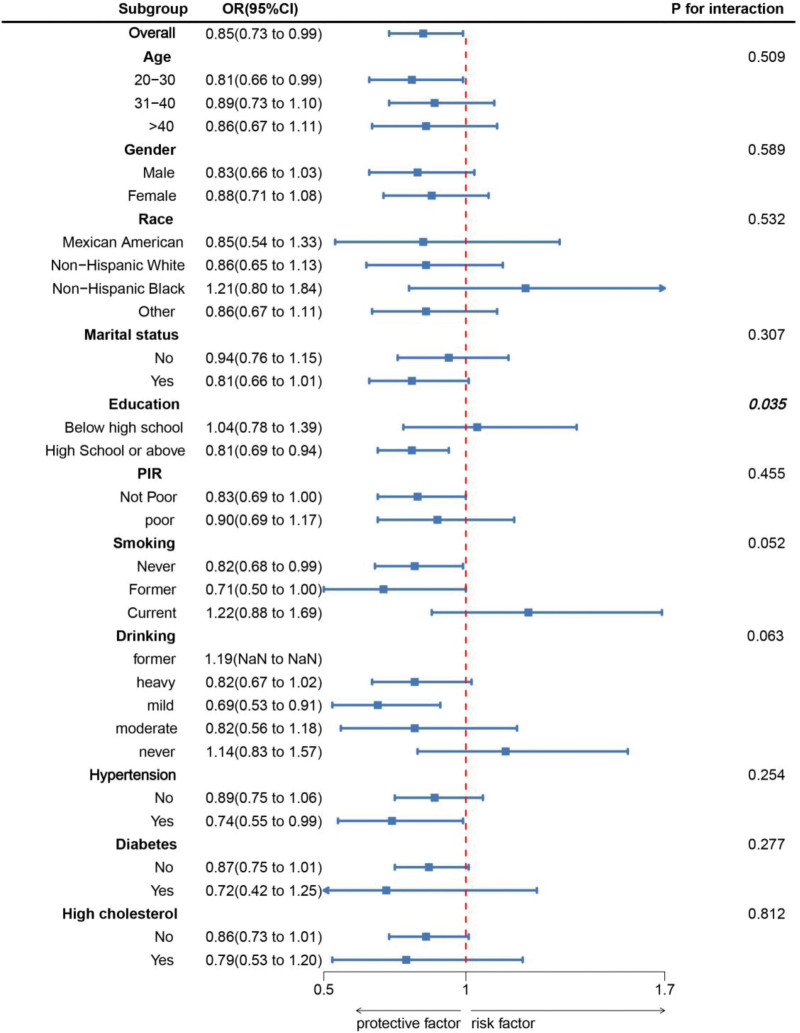
Subgroup analysis between PHDI and muscular dystrophies. ORs were calculated as per 10-unit increase in PHDI. Analyses were adjusted for age, gender, education level, marital, PIR, race, smoking, drinking, hypertension, diabetes, and high cholesterol. OR = odds ratio, PHDI = planetary health diet index, PIR = family poverty income ratio.

As shown in Figure 1, there is a significant negative correlation between PHDI and the prevalence of MDs (overall *P* = .028; nonlinear *P* = .976). Subgroup analysis indicates a consistent relationship between PHDI and the prevalence of MDs across most subgroups (Fig. 2).

### 3.3. Phenotypic age and risk of MDs

Table 2 illustrates the relationship between MDs and phenotypic age. In Model 3, after controlling for all variables, the odds of MDs increased by 33% in the third quartile (T3) compared to the first quartile (T1) [OR = 3.62, (95% CI: 1.20–10.90)]. When phenotypic age is considered as a continuous variable, the positive correlation between MDs and phenotypic age remains statistically significant (OR = 1.07, 95% CI: 1.01–1.14). The results of Model 1 and Model 2 are consistent with these findings.

### 3.4. Association of PHDI and phenotypic age

After adjusting for each covariate, Table 3 shows a significant statistical correlation between PHDI and phenotypic age (β = ‐0.31, 95% CI: −0.53 to −0.10, *P* = .007).

**Table 3:** Multivariate linear regression of PHDI and phenotypic age.

	β	95% CI	*P*-value
PHDI (phenotypic age)	‐0.31	(-0.53 to −0.10)	.007

Adjusted for age, gender, education level, marital, PIR, race, smoking, drinking, hypertension, diabetes, and high cholesterol.

PHDI = planetary health diet index, PIR = ratio of family income to poverty.

### 3.5. Mediation effect

**Figure 3.**
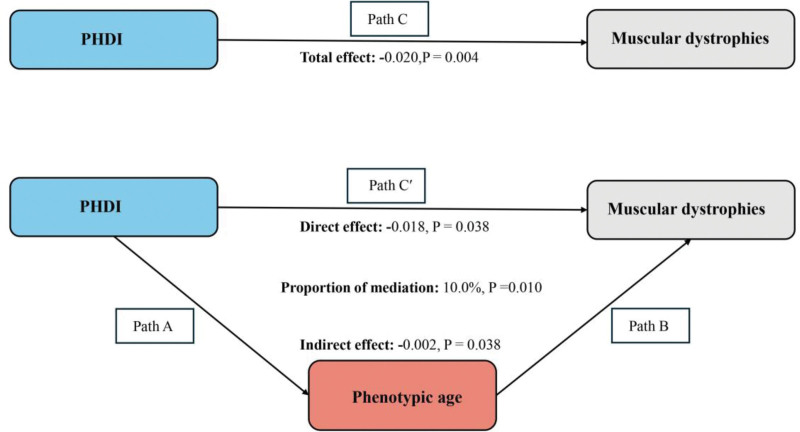
Schematic diagram of the mediation effect analysis. Path C indicates the total effect; path C′ indicates the direct effect. The indirect effect is estimated as the multiplication of paths A and B (path A*B). The mediated proportion is calculated as indirect effect/(indirect effect + direct effect) × 100%. Analyses were adjusted for age, gender, education level, marital, PIR, race, smoking, drinking, hypertension, diabetes, and high cholesterol. PHDI = planetary health diet index, PIR = family poverty income ratio.

The above analysis indicates that our study meets the requirements for conducting a mediation analysis. After adjusting for all covariates, we observed the mediating effect of phenotypic age (Fig. 3) (indirect effect = ‐0.002, *P* = .038; direct effect = ‐0.018, *P* = .038). Therefore, phenotypic age can be regarded as a mediating factor in the association between PHDI and MDs.

## 4. Discussion

This study, based on data from the NHANES between 2011 and 2018, explored the relationship between the PHDI and MDs, as well as the mediating role of phenotypic age. The results demonstrated a significant negative correlation between PHDI scores and the risk of MDs, and this association is partially mediated by phenotypic age. These findings align with the potential benefits of healthy and sustainable dietary patterns proposed by the EAT-Lancet Commission.^[8]^

The Mediterranean diet is a plant-based dietary pattern that primarily uses olive oil as its main fat source.^[21]^ Research has shown that the Mediterranean diet has protective effects against risk factors associated with age-related diseases.^[22]^ Over the past few decades, the Mediterranean diet has been promoted globally as one of the healthiest dietary patterns.^[22]^ Several studies have indicated that the Mediterranean diet can slow the aging process, increase muscle mass, enhance muscle strength, and improve physical function, thereby providing certain benefits for MDs.^[23,24]^ Our study further confirms the potential benefits of a plant-based diet for muscle health while offering a more comprehensive assessment method using the PHDI as an indicator of dietary quality. Additionally, our findings resonate with the research by Campbell et al^[25]^ regarding the relationship between dietary protein intake and muscle mass in older adults. However, our study goes a step further by emphasizing the importance of plant-based protein sources. This is particularly significant because animal protein has traditionally been regarded as crucial for maintaining muscle mass. Our results suggest that a well-designed plant-based diet may be equally effective, if not more beneficial, for overall health and environmental sustainability. Research has found that dietary quality is associated with long-term changes in muscle mass and function.^[26]^ By introducing phenotypic age as a mediating factor, our study provides new insights into the potential mechanisms underlying this relationship. This approach allows us to explore how diet can influence muscle health by affecting biological aging processes, thereby offering a more integrated understanding framework.

The PHDI encourages the intake of more fruits, vegetables, and whole grains, which are rich in antioxidants and anti-inflammatory compounds that may help reduce chronic inflammation and oxidative stress, thereby protecting muscle tissue. Chronic inflammation and oxidative stress are considered key factors in muscle degeneration.^[27]^ Chronic inflammatory responses can lead to an imbalance in muscle protein synthesis and degradation, while oxidative stress increases the production of reactive oxygen species, which can damage muscle cells and lead to declines in muscle mass and strength. Vitamin C is a vital antioxidant, and scientific research has shown that it can reduce exercise-induced muscle damage and promote muscle recovery.^[28]^ Plant-based foods are abundant in antioxidants, including vitamin C, vitamin E, carotenoids, and polyphenols. These compounds can neutralize free radicals and reduce oxidative damage, thereby protecting muscle cells. Additionally, plant-based diets may mitigate this process by lowering levels of inflammatory markers such as CRP and interleukin-6.^[29]^ Polyphenolic compounds, omega-3 fatty acids, carotenoids, and flavonoids found in plant-based foods are also recognized for their anti-inflammatory properties.^[30]^ Omega-3 fatty acids, particularly EPA and DHA, have a positive impact on musculoskeletal health. Supplementing with omega-3 fatty acids can improve muscle mass and volume, enhance muscle strength and physical performance, and positively influence muscle protein synthesis.^[31]^

In recent years, the functions of gut microbiota and their metabolites have been extensively explored. There is now substantial evidence supporting the existence of the gut–microbiome–muscle axis,^[32,33]^ and plant-based diets may influence muscle health by improving the composition of gut microbiota. Plant-based diets are typically rich in prebiotics and dietary fiber, which promote the growth of beneficial bacteria in the gut, thereby helping to maintain a balanced and healthy gut microbiome.^[34]^ Moreover, specific nutrients in plant-based diets, such as arginine and indigestible carbohydrates, can be fermented by gut microbiota to produce metabolites beneficial to gut health.^[35]^ Gut microbiota can influence muscle quality, strength, and endurance through various mechanisms. Firstly, important metabolites produced by the fermentation of gut microbiota, such as short-chain fatty acids (SCFAs), are believed to play a crucial role in maintaining muscle quality. SCFAs can promote glycogen synthesis in muscles^[36]^ and increase the uptake and oxidation of fatty acids, thereby enhancing metabolic efficiency in muscles. SCFAs can also enhance glucose uptake and glycogen synthesis in muscles by promoting the secretion of hormones like glucagon-like peptide-1.^[37]^ Additionally, gut microbiota play significant roles in energy metabolism,^[38]^ inflammatory responses,^[39]^ and oxidative stress,^[40]^ which collectively impact muscle health.

Elderly individuals diagnosed with muscle degeneration often have lower intakes of essential nutrients such as selenium, calcium, magnesium, iron, and sodium.^[41]^ This may be related to factors such as their dietary habits, decreased absorption capacity, and chronic diseases. Plant-based diets are rich in various essential nutrients that are necessary for maintaining muscle health. Magnesium is involved in energy metabolism processes and helps maintain normal muscle contraction and relaxation. Studies have shown that muscle performance in the elderly is positively correlated with serum magnesium levels.^[42]^ Calcium ions play a crucial role in the transmission between muscle fibers, which is essential for maintaining muscle strength and responsiveness.^[43]^ Additionally, calcium ions can activate calcium/calmodulin-dependent protein kinases,^[44]^ which are involved in regulating glycogen breakdown and fatty acid oxidation, thereby playing an important role in muscle metabolism. Systematic reviews have emphasized the potential benefits of selenium in preventing and managing muscle degeneration in the elderly.^[45]^ Selenium is an antioxidant that helps protect muscle cells from oxidative stress damage. In summary, appropriately adjusting dietary structures and supplementing key nutrients may be effective strategies for improving muscle health in the elderly.

An interesting finding of this study is the significant interaction between PHDI and education level, suggesting that the inverse association between diet quality and MDs was primarily evident among individuals with at least a high school education, but not among those with lower educational attainment. Several mechanisms may explain this disparity. First, individuals with higher education levels generally have greater health literacy and nutritional knowledge, which may facilitate better understanding and adherence to dietary recommendations reflected by higher PHDI scores. Second, higher educational attainment is often associated with improved socioeconomic status, which may enable greater access to high-quality, nutrient-dense foods consistent with sustainable dietary patterns. In contrast, individuals with lower education levels may face structural barriers, including limited access to healthy foods, financial constraints, and reduced awareness of dietary guidelines, potentially attenuating the beneficial association between diet quality and muscle health. Furthermore, this finding highlights the importance of considering social determinants of health when evaluating diet–health relationships. The differential association across education strata suggests that improving diet quality alone may not be sufficient to reduce disparities in muscle health without addressing underlying socioeconomic and educational inequalities. From a public health perspective, targeted nutritional interventions and educational strategies may be particularly necessary for populations with lower educational attainment to enhance the effectiveness of dietary recommendations.

Our study is the first to reveal the mediating role of phenotypic age in the relationship between the PHDI and muscle degeneration. As a marker of biological aging, phenotypic age reflects overall health status.^[46]^ We found that PHDI is associated with lower phenotypic age, which in turn is linked to a reduced risk of muscle degeneration. Thus, a plant-based diet may protect muscle health by slowing the biological aging process. The mediating effect of phenotypic age may reflect multiple underlying mechanisms. Phenotypic age may capture age-related physiological changes, such as alterations in hormone levels (e.g., decreases in testosterone, growth hormone, and insulin-like growth factor-1). Research indicates that changes in multiple hormone levels collectively influence muscle quality and function, leading to the development of muscle degeneration.^[47]^ Furthermore, various components of plant-based diets can improve mitochondrial function.^[48]^ Studies have shown that mitochondrial dysfunction is a key factor in accelerated aging.^[49]^ Mitochondria, as the central hub of cellular energy metabolism, are crucial for muscle health. A recent review article^[50]^ discussed the role of mitochondria in maintaining muscle health and preventing age-related declines in muscle function, emphasizing the importance of nutritional interventions in improving mitochondrial function to sustain muscle health.

The main strength of this study lies in its use of a large, representative NHANES dataset, which enhances the generalizability of the results. The rigorous sampling and standardized data collection procedures of NHANES ensure the high quality and reliability of the data. Additionally, we employed advanced statistical methods, including mediation analysis, which allowed us to explore the complex relationships among the PHDI, phenotypic age, and muscle degeneration. However, we also acknowledge some limitations in the study. First, the cross-sectional design limits our ability to infer causality. While we identified associations among PHDI, phenotypic age, and muscle degeneration, we cannot determine the temporal order or causal direction of these relationships. Additionally, phenotypic age was derived from biomarkers such as CRP, albumin, and creatinine, which are themselves influenced by muscle mass and muscle condition; therefore, a potential reverse-causation pathway may exist whereby low muscle mass affects phenotypic age, rather than the assumed direction, which may bias the observed mediation effect and further limits causal interpretation. Importantly, due to the cross-sectional design of the NHANES data, causal mediation inference is not possible; therefore, the estimated mediation proportion should be interpreted as a descriptive decomposition of association rather than evidence of a causal mechanism. Second, although NHANES data are of high quality, self-reported dietary information may be subject to recall bias. Participants may underestimate or overestimate their intake of certain foods, especially considering that dietary data are collected using a 24-hour recall method, which may not fully reflect long-term dietary patterns. Third, while we controlled for several potential confounding factors, there may still be unmeasured confounders that influence the results. For instance, we cannot completely rule out the effects of genetic factors or certain environmental exposures. Finally, although PHDI is a comprehensive measure of dietary quality, it may not capture all dietary factors related to muscle health. For example, certain specific nutrients or food combinations may have unique effects on muscle health but may not be adequately reflected in the PHDI. Although NHANES is a nationally representative survey with rigorous quality control, the assessment of MDs was only available in the 2011 to 2018 survey cycles. As a result, more recent NHANES data could not be incorporated into the analysis, which may limit the temporal generalizability of our findings. Nonetheless, these survey cycles represent the most comprehensive and reliable data currently available for investigating MDs at the population level in the United States.

Our analysis’s definition of muscular dystrophy was derived from earlier NHANES studies, which might not accurately reflect the clinical diagnostic standards. Despite being in line with earlier research, this method may result in misclassification and restrict how broadly our results may be applied. Furthermore, there may still be some conceptual uncertainty because the criteria of muscular dystrophy and sarcopenia share characteristics related to functional decline and muscle weakening. As a result, our results should be interpreted cautiously, and additional research with clinically recognized muscular dystrophy diagnosis criteria is necessary to validate and expand upon our findings. Future studies incorporating clinically confirmed diagnoses, genetic data, or longitudinal neuromuscular assessments are warranted to validate and extend our findings.

In the future, we will further deepen the research on the relationships among the PHDI, phenotypic age, and muscle degeneration. First, conducting longitudinal studies can help establish causal relationships among these factors and observe the long-term effects of dietary patterns on muscle health. Second, designing randomized controlled trials can directly assess the effects of PHDI interventions on improving muscle health, providing more robust evidence for clinical practice. In-depth research into how specific components of PHDI affect muscle health may assist in optimizing dietary recommendations. Additionally, integrating omics approaches (such as metabolomics, proteomics, and epigenomics) can reveal the molecular mechanisms underlying these relationships. Exploring the interactions between PHDI and other lifestyle factors (such as physical activity, sleep quality, and stress levels) will aid in developing more comprehensive prevention and intervention strategies. These research directions will provide valuable scientific evidence for improving public health.

## 5. Conclusion

In summary, this study provides new insights into the complex relationships among PHDI, phenotypic age, and muscle degeneration. Our findings emphasize the potential role of a healthy diet in maintaining muscle health and highlight the importance of biological aging as a mediating factor in this relationship. As an innovative assessment tool, PHDI focuses on individual health and considers the environmental impact of dietary choices. The possible association between this index and muscle degeneration offers a new perspective for understanding the relationship between diet and muscle health. Given the significant impact of muscle degeneration on patients’ quality of life and the need for long-term care, it represents an undeniable part of the global disease burden. Including phenotypic age as a mediating factor further enriches the depth of this research. As a measure of biological age, phenotypic age reflects an individual’s actual aging status, which is particularly relevant in today’s increasingly aging society. Additionally, the Planetary Health Diet aims not only to improve human health but also to reduce the negative environmental impacts of dietary practices. By studying the association between PHDI and specific diseases, we can provide scientific evidence to promote sustainable and healthy dietary patterns, thereby improving both human health and the health of the planet’s ecosystems.

## Acknowledgments

We sincerely appreciate the NHANES database for all of the data.

## Author contributions

**Conceptualization:** Xiaomei Lin, Shaoqun Huang, Hongyang Gong.

**Data curation:** Xiaomei Lin, Shaoqun Huang, Hongyang Gong.

**Formal analysis:** Xiaomei Lin, Shaoqun Huang, Hongyang Gong.

**Funding acquisition:** Shaoqun Huang.

**Investigation:** Shaoqun Huang, Hongyang Gong.

**Methodology:** Shaoqun Huang, Hongyang Gong.

**Project administration:** Shaoqun Huang, Hongyang Gong.

**Resources:** Shaoqun Huang, Hongyang Gong.

**Software:** Shaoqun Huang, Hongyang Gong.

**Supervision:** Shaoqun Huang, Hongyang Gong.

**Validation:** Shaoqun Huang, Hongyang Gong.

**Visualization:** Shaoqun Huang, Hongyang Gong.

**Writing – review & editing:** Shaoqun Huang.

**Writing – original draft:** Hongyang Gong.






