# Supplementary material for: The association between the planetary health diet index (PHDI) and muscular dystrophies: A mediating role of phenotypic age
Source: Medicine (Baltimore). 2026 May 8;105(19):e48612. doi: 10.1097/MD.0000000000048612 (PMC13166471; doi:10.1097/MD.0000000000048612)
Supplement: Supplementary file 1 [file medi-105-e48612-s001.docx]

**Supplementary Material**


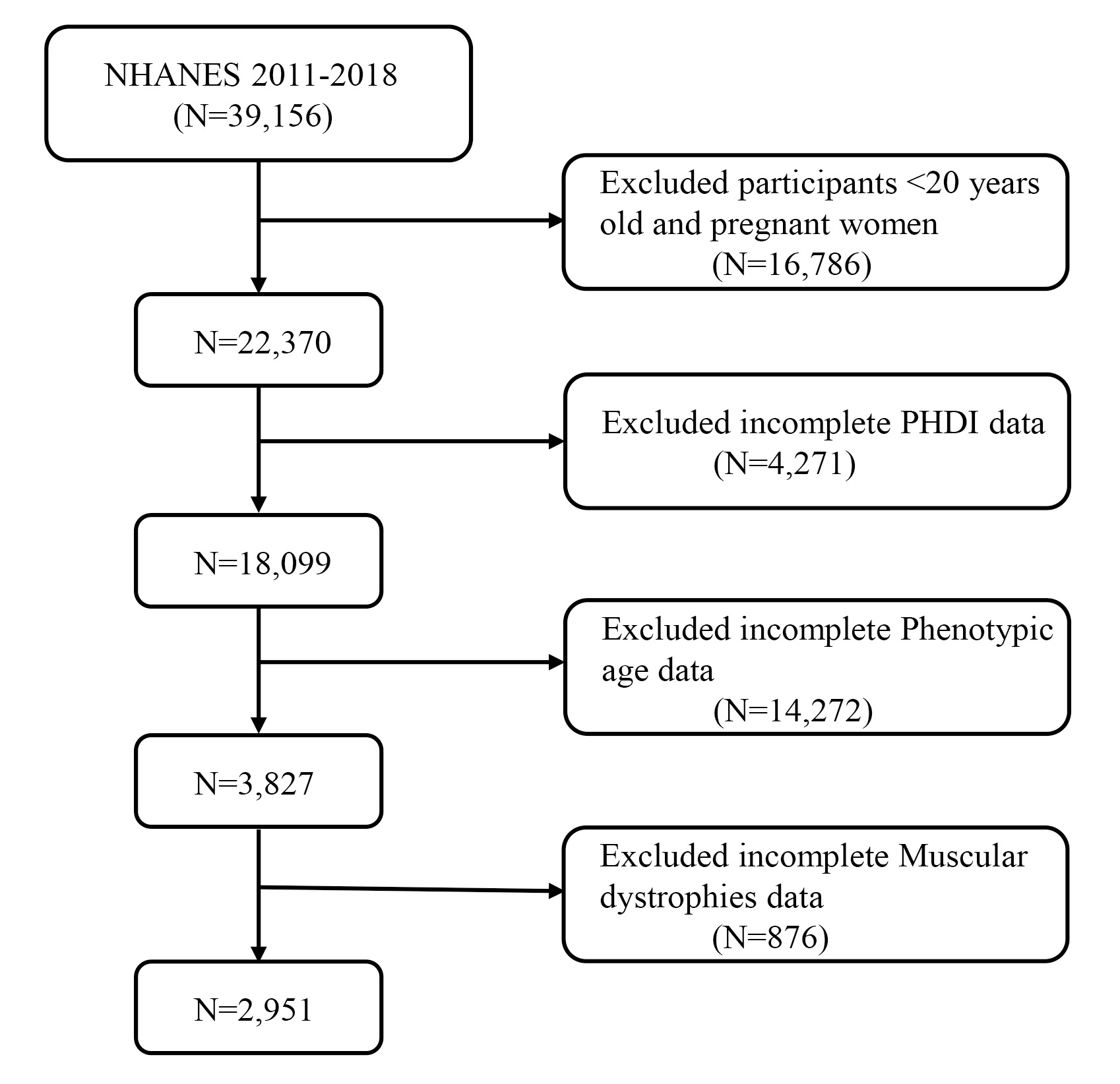


**Figure S1.** A flow diagram of eligible participant selection in the National Health and Nutrition Examination Survey.

Abbreviation: PHDI, Planetary Health Diet Index.
