# Supplementary material for: The association between the planetary health diet index (PHDI) and muscular dystrophies: A mediating role of phenotypic age
Source: Medicine (Baltimore). 2026 May 8;105(19):e48612. doi: 10.1097/MD.0000000000048612 (PMC13166471; doi:10.1097/MD.0000000000048612)
Supplement: Supplementary file 2 [file medi-105-e48612-s002.docx]

**Supplementary Material**

| **Table S1.** Scoring criteria for the Planetary Health Diet Index (PHDI). | | |
| --- | --- | --- |
| **Dietary component** | **Category minimum score** (0 points) | **Category maximum score** (10 points) |
| ***Adequacy components*** | | |
| Whole grains^1^ | 0 grams | ≥ 75 grams for women  ≥ 90 grams for men |
| Whole fruits (excludes fruit juice) | 0 grams | ≥ 200 grams |
| Non-starchy vegetables | 0 grams | ≥ 300 grams |
| Nuts and seeds | 0 grams | ≥ 50 grams |
| Legumes |  |  |
| Non-soy legumes^2,3^ | 0 grams | 100 grams |
| Soybean/ soy foods^2,3^ | 0 grams | 50 grams |
| Unsaturated oils | 0% of total energy intake | ≥ 10% of total energy intake |
| ***Moderation components*** | | |
| Starchy vegetables | ≥ 200 grams | ≤ 50 grams |
| Dairy^4^ | ≥ 4.08 cup-equivalents | ≤ 1.02 cup-equivalents |
| Red and processed meat | ≥ 300 grams | ≤ 14 grams |
| Poultry | ≥ 58 grams | ≤ 29 grams |
| Eggs | ≥ 120 grams | ≤ 12 grams |
| Fish | ≥ 50 grams | ≤ 15 grams |
| Saturated oils and *trans* fats | ≥ 21% of total energy intake | ≤ 3.5% of total energy intake |
| Added sugar and fruit juice | ≥ 25% of total energy intake | ≤ 5% of total energy intake |
| ^1^Thresholds were based on the midpoint of the recommended range listed in EAT-Lancet Commission Scientific Report.  ^2^Grams per day calculated from dry weight.  ^3^To calculate the score for the legumes component, the nonsoy and soy subcomponents were each weighted at 0.5.  ^4^In FPED, 1 serving of dairy is equal to 245 g of whole-milk or derivative equivalent. In the EAT-Lancet report, scores were assigned ≤250 g whole-milk or derivative equivalent for the maximum score or ≥1000 g whole-milk or derivative equivalent for the minimum score.  Note: The above dietary components were obtained from the Food Pattern Equivalence Database (FPED) of the 24-hour dietary recall data(https://www.ars.usda.gov/northeast-area/beltsville-md-bhnrc/beltsville-human-nutrition-research-center/food-surveys-research-group/docs/fped-databases/). | | |
