# Supplementary material for: The association between the planetary health diet index (PHDI) and muscular dystrophies: A mediating role of phenotypic age
Source: Medicine (Baltimore). 2026 May 8;105(19):e48612. doi: 10.1097/MD.0000000000048612 (PMC13166471; doi:10.1097/MD.0000000000048612)
Supplement: Supplementary file 3 [file medi-105-e48612-s003.docx]

**Supplementary Material**

**Table S2.** Definition of variables involved in this study.

| Variables | Description in NHANES |
| --- | --- |
| Age | Divided into three groups: 20-40 years old, 41-60  years old, >60 years old |
| Gender | Male and Female |
| Race | Mexican American, Non-Hispanic Black, Non-Hispanic White, Other Race |
| Educational level | Below high school, High School or above |
| Marital status | Yes: Married/Living with partner |
| PIR | Poor: <1.3; Not Poor:>=1.3 |
| Smoking | Smoking status was grouped into never smoker (defined as <100 cigarettes in a lifetime), current smoker (defined as ≥100 cigarettes in a lifetime), and former smoker (defined as ≥100 cigarettes and had quit smoking) |
| Drinking | heavy drinking (≥4 drinks/day for men, ≥3 drinks/day for women, or ≥5 days of drinking in a month),  moderate drinking (≥3 drinks/day for men, ≥2 drinks/day for women, or ≥2 days of drinking in a month),  mild drinking (≤2 drinks/day for men, ≤1 drink/day for women, and ≥12 drinks in a year),  and never-drinking (total number of drinks in a year <12, and dietary alcohol content of 0%) |
| Diabetes | Diabetes was defined as a history of previous diabetes, HbA1c level ≥6.5%, or fasting blood glucose level ≥126 mg/dL |
| Hypertension | The diagnostic criteria consist of self-reported hypertension history, the utilization of antihypertensive medication, a systolic blood pressure (SBP) ≥ 140mmHg, or a diastolic blood pressure (DBP) ≥ 90mmHg |
| High cholesterol | Participants were asked whether they had high cholesterol |

PIR, Ratio of family income to poverty.
